# Supplementary material for: The ratio of expansion to compression: A new measure of lifespan disparity
Source: PLoS One. 2020 Dec 14;15(12):e0243482. doi: 10.1371/journal.pone.0243482 (PMC7735627; doi:10.1371/journal.pone.0243482)
Supplement: Attachment — Submitted filename: Review.pdf [file pone.0243482.s002.pdf]

Thank your manuscript. The topic addressed is very interesting and will contribute to understanding the dynamics of changes in lifespan disparity following a decline in mortality at younger (older) ages.

General comments:

- the manuscript is well written and structured,
- it is in general easy too follow,
- I would suggest a few modifications that in my opinion will make it easier to read and hence will keep the reader interested.
- The Decomposition of the change over time of the REC will be easier and better understood with a numerical example. Therefore, I suggest taking the Illustrative example in the Result section and added it to the Decomposition of the change over time of the REC section; a structure that will combine the equations and the numeric example for instance. The current structure results in too many repetitions. Some of the properties discussed in the "Decomposition of the change over time of the REC" section are again mentioned in the "Illustrative example". We tend to skip the Illustrative example. It's a pity because the example allows a easier understanding of the dynamics between the REC, the compression component, the expansion component and their changes over time. Figure 1, could then reflect g(x) and $\rho(x)$ for Russia and Japan for each calendar year. Such as figure would be more interesting given that it reflects an increase in mortality at younger ages (Russia) and a decline in mortality at older ages (Japan). Figure 1 upper and lower panel add vertical lines for the $a^\dagger$ and explain the decline/increase in mortality in relation to $a^\dagger$.
- Simulation section is very interesting. However, a table (rows: parameters x columns: scenarios) showing the Siler's model parameters for each of the three scenarios will facilitate the reading and comprehension of the different models used. The text is a bit hard to understand which parameter corresponds to which scenario(s).
- When I read the 1st paragraph of the Simulation section, I was expecting to see how $a^\dagger$ changes under the three scenarios and a result that will show under which scenario $a^\dagger$ increases most.
- You also mention "faster progress above threshold age $a^\dagger$". But what is the value of this threshold age at baseline? And how did the $a^\dagger$ changed under the 3 scenarios. Given that $e^\dagger$, $e_c^\dagger$, $e_e^\dagger$, and REC depend on $a^\dagger$ it is important to document its changes under the different scenarios.
- What do the simulated years exactly represent? The way I was expecting the simulations to go is as follow: (1) I have $\mu(x)$ function at t_0 and from t_0 to t_1 mortality declined faster at younger than older ages (2) Look at how this decline affected $e^\dagger$, $e_c^\dagger$, $e_e^\dagger$ and REC. I am guessing that you have repeated this process several times, given the trends in Figure 4. If this is the case, then Figure 4 green line shows trends in $e^\dagger$, $e_c^\dagger$, $e_e^\dagger$, and REC if mortality declines faster below $a^\dagger$ year after year after year.

Here are my more detailed comments:

- p. 3, line 46: define entropy
- p. 3, line 58: "In a country with increasing younger adult mortality (because of wars or political distress), the compression component can rise and increase lifespan disparity." My understanding is that compression component declines (i.e. deaths are less compressed) if

mortality at younger adult ages increases. I think here you mean the “compression component declines” or compression “component becomes less important”.

- p. 5, line 89: “entropy less than one”. On p. 3, line 46 “entropy bigger than one” .
- p. 8, line 151: “The expansion component can …”, confusing. It seem that in the sentence above you say the opposite.
- p. 9, line 175: “g(x,t) indicates how efficiently a decrease in …”. What do you mean by efficiently.
- p.9 line 186: “low efficiency”. Explain, why is it considered low efficiency? How much the $e^{\dagger}$ needs to change in order for the old-age mortality decline to be considered efficient?
- p. 10: line 196-197: “(5) represents the decrease in the compression component” … “and the second the increase in the expansion component”. Why automatically decrease and increase? You previously mentioned, that the compression component can decline under particular mortality conditions. Why not changes in the compression and expansion component.
- p. 14 line 287-288: Highlight that the REC is under 1 in Figure 3. The value of the REC is important as it is this value that indicates which component is stronger. Important to mention it when describing the Figure.
- p.15 and p.16: again mention that the REC is below 1 and what does this mean (i.e. REC below 1 indicates that the compression is stronger than the expansion) when talking about Figures 4 and 5. An increase in the REC towards 1, indicates that the expansion component is increasing. You do not mention this when describing Figure 4 and 5.
- Discussion section: I would have liked some possible explanations on why the compression component is always bigger than the expansion component, for both cohorts and periods. Do you think that this is due to the fact that $a^{\dagger}$ depends on life expectancy at age x ($e(x,t)$ appears in the $g(x,t)$function?) Could this be a limitation of the indicator because $e(x,t)$ is highly sensitive to mortality changes at younger than older ages? Also, how does $a^{\dagger}$,$e^{\dagger}$, $e_c^{\dagger}$, $e_e^{\dagger}$, and REC change with different values of x, i.e. $x = 0$, $x = 10$, $x = 20$? I assume that for the entire manuscript you chose $x = 0$.
