# Supplementary material for: The ratio of expansion to compression: A new measure of lifespan disparity
Source: PLoS One. 2020 Dec 14;15(12):e0243482. doi: 10.1371/journal.pone.0243482 (PMC7735627; doi:10.1371/journal.pone.0243482)
Supplement: Attachment — Submitted filename: Review_PLOSONE.pdf [file pone.0243482.s003.pdf]

Review of manuscript PONE-D-20-25812:

The ratio of expansion to compression: A new measure of lifespan disparity

Review

The manuscript introduces a new measure of lifespan disparity – the ratio of expansion to compression (REC) – that can shed novel insights into the analysis of lifespan inequality within human populations. The REC measure distinguishes the opposing effects of mortality changes on the compression and expansion of mortality. Separate lifespan disparity measures are computed for the two components by splitting $e^\dagger$ before and after the threshold age, and REC is computed as the ratio of the two measures. The authors apply the new measure (i) in a case study, (ii) in simulation analysis, and (iii) to a series on human populations from both the period and cohort perspective, and they compare the new measure with the traditional lifespan disparity.

I found the manuscript and the proposed methodology interesting. This is one among the first attempts to analyse lifespan inequality by studying the opposing effects of expansion and compression, and I praise the authors for their contribution.

However, I have some main comments and several smaller suggestions to the authors that could help them to improve the manuscript. I elaborate on these in the following.

Main Comments

- Interpretation of changes in REC. Throughout the manuscript, you consistently (and correctly, in my opinion) interpret an increase in REC as an increasing importance of the expansion component with respect to the compression one (see e.g. lines 244, 289, 319). However, when you introduce the REC measure, you write "*a decrease in the REC, regardless as to whether it is above or below one, implies that life chances are distributed more equally (and vice versa)*" (lines 131–132). I am not convinced about this second definition. From your empirical findings (especially from the period perspective), we see that $e^\dagger$ and REC very often moved in opposite directions. I think that the observed increases in REC do not imply lower equality, but rather that the expansion component has been driving the reduction in $e^\dagger$. As such, I find your interpretation of REC at lines 131–132 confusing, and I suggest to remove it and keep only the interpretation at lines 133–134 (i.e. the increasing/decreasing importance of the expansion component wrt the compression one).
- Illustrative example. While this is really interesting and provides a great illustration to your measure, it would be equally interesting and important to provide a second complementary example, where lifespan disparity **decreases** and we observe two different changes in the REC. This is because practically all scenarios of mortality changes analysed in the Section "Empirical REC" and simulations deal with a decrease of $e^\dagger$.
- Codes and reproducibility. Readers would benefit from having the opportunity of using the newly proposed measure in their analysis. As such, I suggest to the authors to provide the

routines developed for this study in the Supplementary Material of the article or in a publicly available repository.

Minor comments

- Line 46: *life table entropy greater than one*. I believe it should be lower than one, as correctly reported in line 89.
- Lines 55-56: "*smaller lifespan disparity is not always favourable*". Since your motivating example in the following lines (56-69) deals with increasing lifespan disparity, I suggest rephrasing/ adding to the sentence "an increase in lifespan disparity is not always unfavourable" for consistency. Otherwise, you could leave the expression as it is and change the motivating example to deal with a reduction in lifespan disparity, which could be positive or negative depending on the changes in mortality.
- Lines 113-115: "*..., the threshold age is located near the age of life expectancy and is generally 2 years or so lower than life expectancy from the 1950s onwards [8].*" The value of the threshold age depends on which lifespan disparity measure we consider, so that greater or lower differences with life expectancy can be observed when using different measures (see, e.g., Aburto et al., 2020, Supplementary Material p.14). I suggest the authors to specify in their sentence that they refer to the threshold age **of lifespan disparity**.
- Line 140: "*Lifespan disparity can increase in two cases*". Similarly to my main comment #2 on the illustrative example, since most of your empirical findings relate to the case of lifespan disparity reductions, it would be interesting to add a complementary paragraph where you describe that lifespan disparity can **decrease** in two cases, and how the REC can change in these cases.
- Line 178: "*1960s, 1980s and 2000s*". In Figure 1, you report the years 1960, 1980 and 2010. You should then adjust the text or the legend in Figure 1 according to which data you are using.
- Lines 196-197: "*The first term on the right-hand side of (5) represents the decrease in the compression component and the second the increase in the expansion component*". For this sentence to be true, we should be analysing the case of mortality decline. As such, I suggest the authors to specify this. An even better solution would be to replace the words "decrease" and "increase" with the word "change".
- Line 200: there is a minus missing in the formula, i.e. the formula should read:

  $$\dot{e}^{\dagger}(t) = 0 \iff \dot{e}^{\dagger}_{e}(t) = -\,\dot{e}^{\dagger}_{c}(t)$$

  Note that the second part of the formula is correct, i.e. the ratio of the two quantities is indeed equal to -1.
- Line 209: "*Similar to (7)*". Maybe you mean similar to (6), where you set the change equal to zero?
- Line 218: "*Note that* $e^{\dagger}_{c} > e^{\dagger}_{e}$". This is true for human populations in most recent years, but it needs not be the case. I would suggest the author to specify this point.
- Line 222: "*greater than* $e^{\dagger}_{c}$". According to my previous remark of the missing minus in Eq. (6), it should be "greater than $-\,e^{\dagger}_{c}$".
- Figure 2: please report the REC value in the two years, similarly to what you have done for $e^{\dagger}$.

- Line 266: please cite the paper of Siler (1979).
- Line 341: "*The REC remained constant until the 1900s–1930s and then rapidly decreased*". I think you meant that it rapidly increased.
- References: the following two important works in this area should be cited in your manuscript: Shkolnikov et al. (2003); Aburto et al. (2019)

References

Aburto, J. M., J.-A. Alvarez-Martínez, F. Villavicencio, and J. W. Vaupel (2019, jul). The threshold age of the lifetable entropy. *Demographic Research 41*, 83–102.

Aburto, J. M., F. Villavicencio, U. Basellini, S. Kjærgaard, and J. W. Vaupel (2020, feb). Dynamics of life expectancy and life span equality. *Proceedings of the National Academy of Sciences 117*(10), 5250–5259.

Shkolnikov, V. M., E. E. Andreev, and A. Z. Begun (2003). Gini coefficient as a life table function: computation from discrete data, decomposition of differences and empirical examples. *Demographic Research 8*, 305–358.

Siler, W. (1979). A competing-risk model for animal mortality. *Ecology 60*(4), 750–757.
