# Supplementary material for: The ratio of expansion to compression: A new measure of lifespan disparity
Source: PLoS One. 2020 Dec 14;15(12):e0243482. doi: 10.1371/journal.pone.0243482 (PMC7735627; doi:10.1371/journal.pone.0243482)
Supplement: Attachment — Submitted filename: Response to Reviewers.docx [file pone.0243482.s004.docx]

## Reviewer A:

Review of manuscript PONE-D-20-25812:

***The ratio of expansion to compression: A new measure of lifespan disparity***

**Review**

The manuscript introduces a new measure of lifespan disparity – the ratio of expansion to compression (REC) – that can shed novel insights into the analysis of lifespan inequality within human populations. The REC measure distinguishes the opposing effects of mortality changes on the compression and expansion of mortality. Separate lifespan disparity measures are computed for	the two components by splitting *e^†^* before and after the threshold age, and REC is computed as	the ratio of the two measures. The authors apply the new measure (i) in a case study, (ii) in simulation analysis, and (iii) to a series on human populations from both the period and cohort perspective, and they compare the new measure with the traditional lifespan disparity.

I found the manuscript and the proposed methodology interesting. This is one among the first attempts to analyse lifespan inequality by studying the opposing effects of expansion and compression, and I praise the authors for their contribution.

However, I have some main comments and several smaller suggestions to the authors that could help them to improve the manuscript. I elaborate on these in the following.

**Main Comments**

- Interpretation of changes in REC. Throughout the manuscript, you consistently (and correctly, in my opinion) interpret an increase in REC as an increasing importance of the expansion component with respect to the compression one (see e.g. lines 244, 289, 319). However, when you introduce the REC measure, you write “*a decrease in the REC, regardless as to whether it is above or below one, implies that life chances are distributed more equally (and vice versa)*” (lines 131–132). I am not convinced about this second definition. From your empirical findings (especially from the period perspective), we see that *e*^†^ and REC very often moved in opposite directions. I think that the observed increases in REC do not imply lower equality, but rather that the expansion component has been driving the reduction in *e*^†^. As such, I find your interpretation of REC at lines 131–132 confusing, and I suggest to remove it and keep only the interpretation at lines 133–134 (i.e. the increasing/decreasing importance of the expansion component wrt the compression one).

-- Great thanks for pointing out this. We agree that the interpretation of REC at lines 131-132 is inconsistent with other interpretations of REC. Following your suggestions, we removed it so as to have a consistent understanding of changes in REC.

- Illustrative example. While this is really interesting and provides a great illustration to your measure, it would be equally interesting and important to provide a second complementary example, where lifespan disparity **decreases** and we observe two different changes in the REC. This is because practically all scenarios of mortality changes analysed in the Section “Empirical REC” and simulations deal with a decrease of *e*^†^.

-- This idea is really interesting and will be helpful for a better understanding of how lifespan disparity changes. Hence, we added an example for the decreasing *e*^†^: Belgium men in 1960-1965 and England & Wales men in 2000-2005 both experienced the decrease of *e*^†^, but with opposite composition of changes in $e_{c}^{\dagger}$and $e_{e}^{\dagger}$. Moreover, the REC increased for Belgium men but decreased for England & Wales men.

- Codes and reproducibility. Readers would benefit from having the opportunity of using the newly proposed measure in their analysis. As such, I suggest to the authors to provide the routines developed for this study in the Supplementary Material of the article or in a publicly available repository.

-- We are glad to share the codes with the community. The codes are available online at GitHub.

**Minor comments**

- Line 46: *life table entropy greater than one*. I believe it should be lower than one, as correctly reported in line 89.

-- Thanks for pointing out this. We corrected it.

- Lines 55-56: “*smaller lifespan disparity is not always favourable*”. Since your motivating ex- ample in the following lines (56-69) deals with increasing lifespan disparity, I suggest rephrasing/ adding to the sentence “an increase in lifespan disparity is not always unfavourable” for consistency. Otherwise, you could leave the expression as it is and change the motivating example to deal with a reduction in lifespan disparity, which could be positive or negative depending on the changes in mortality.

-- Your rephrasing of this sentence is very accurate. We adopt it in our revision.

- Lines 113-115: “*. . . , the threshold age is located near the age of life expectancy and is generally 2 years or so lower than life expectancy from the 1950s onwards [8].*” The value of the threshold age depends on which lifespan disparity measure we consider, so that greater or lower differences with life expectancy can be observed when using different measures (see, e.g., Aburto et al., 2020, Supplementary Material p.14). I suggest the authors to specify in their sentence that they refer to the threshold age **of lifespan disparity**.

-- Many thanks. In revision, we specified that the threshold age is for lifespan disparity.

- Line 140: “*Lifespan disparity can increase in two cases* ”. Similar to my main comment #2 on the illustrative example, since most of your empirical findings relate to the case of lifespan

disparity reductions, it would be interesting to add a complementary paragraph where you describe that lifespan disparity can **decrease** in two cases, and how the REC can change in these cases.

-- Thanks for your suggestions. We added a paragraph to discuss the case of the lifespan disparity decrease in the same section.

- Line 178: “*1960s, 1980s and 2000s*”. In Figure 1, you report the years 1960, 1980 and 2010. You should then adjust the text or the legend in Figure 1 according to which data you are

using.

-- Thanks for pointing out this. We correct it.

- Lines 196-197: “*The first term on the right-hand side of (5) represents the decrease in the compression component and the second the increase in the expansion component*”. For this sentence to be true, we should be analysing the case of mortality decline. As such, I suggest

the authors to specify this. An even better solution would be to replace the words “decrease” and “increase” with the word “change”.

-- This is a valid point. Indeed, the word “change” is much better than “decrease” or “increase.”

- Line 200: there is a minus missing in the formula, i.e. the formula should read:

*e*˙^†^(*t*) = 0 ⇐⇒ *e*˙*^†^*e(*t*) = − *e*˙*^†^*c(*t*)

Note that the second part of the formula is correct, i.e. the ratio of the two quantities is indeed equal to -1.

-- You are right. We corrected it in the revision.

- Line 209: “*Similar to (7)*”. Maybe you mean similar to (6), where you set the change equal to zero?

-- Many thanks. We corrected it.

- Line 218: “*Note that e*^†^*_c_ > e*^†^*_e_*”. This is true for human populations in most recent years, but it needs not be the case. I would suggest the author to specify this point.

-- Thanks for reminding us of this. We added a sentence for it.

- Line 222: “*greater than e*^†^*_c_*”. According to my previous remark of the missing minus in Eq. (6), it should be “greater than − *e*^†^*_c_*”.

-- Thanks. We corrected it.

Figure 2: please report the REC value in the two years, similarly to what you have done for

•

*e*^†^.

-- The REC value is added in the figure.

- Line 266: please cite the paper of Siler (1979).

-- Thanks. We added a reference there.

- Line 341: “*The REC remained constant until the 1900s–1930s and then rapidly decreased* ”. I think you meant that it rapidly increased.

-- Thanks for pointing out this. We corrected it.

- References: the following two important works in this area should be cited in your manuscript: Shkolnikov et al. (2003); Aburto et al. (2019)

-- Thanks for reminding us of the two important papers. We cited them in revision.

**References**

Aburto, J. M., J.-A. Alvarez-Mart´ınez, F. Villavicencio, and J. W. Vaupel (2019, jul). The threshold age of the lifetable entropy. *Demographic Research 41*, 83–102.

Aburto, J. M., F. Villavicencio, U. Basellini, S. Kjærgaard, and J. W. Vaupel (2020, feb). Dynamics of life expectancy and life span equality. *Proceedings of the National Academy of Sciences 117* (10), 5250–5259.

Shkolnikov, V. M., E. E. Andreev, and A. Z. Begun (2003). Gini coefficient as a life table function: computation from discrete data, decomposition of differences and empirical examples. *Demographic Research 8*, 305–358.

Siler, W. (1979). A competing-risk model for animal mortality. *Ecology 60* (4), 750–757.

## Reviewer B:

Thank your manuscript. The topic addressed is very interesting and will contribute to understanding the dynamics of changes in lifespan disparity following a decline in mortality at younger (older) ages.

General comments:

- the manuscript is well written and structured,

- it is in general easy too follow,

- I would suggest a few modifications that in my opinion will make it easier to read and hence

will keep the reader interested.

- The Decomposition of the change over time of the REC will be easier and better understood with a numerical example. Therefore, I suggest taking the Illustrative example in the Result section and added it to the Decomposition of the change over time of the REC section; a structure that will combine the equations and the numeric example for instance. The current structure results in too many repetitions. Some of the properties discussed in the “Decomposition of the change over time of the REC” section are again mentioned in the “Illustrative example”. We tend to skip the Illustrative example. It's a pity because the example allows a easier understanding of the dynamics between the REC, the compression component, the expansion component and their changes over time. Figure 1, could then reflect g(x) and for Russia and Japan for each calendar year. Such as figure would be more interesting given that it reflects an increase in mortality at younger ages (Russia) and a decline in mortality at older ages (Japan). Figure 1 upper and lower panel add vertical lines for the and explain the decline/increase in mortality in relation to $a^{\dagger}$.

-- Thank you very much. Inspired by your suggestions, we tried to put the example in the Decomposition section. Moreover, following another Reviewer’s advice, we added the case of the decrease in lifespan disparity. Mathematical relations indeed come with a numerical example can be better understood. Still, we found the example with many numeric results of four countries seems too much for the Decomposition section. The example is thus kept as an independent part. We agree with you that there are some unnecessary repetitions. In revision, we removed some of them and re-phased in the affected parts. Please see the paragraphs marked in yellow color. Accordingly, we re-plotted Figure 1 for the selected populations, in addition to Japanese women. With the figure, the readers could find it easier to understand the change of expansion and compression and the REC. Besides, following your suggestions, we added vertical lines to mark the threshold ages.

- Simulation section is very interesting. However, a table (rows: parameters x columns: scenarios) showing the Siler’s model parameters for each of the three scenarios will facilitate the reading and comprehension of the different models used. The text is a bit hard to understand which parameter corresponds to which scenario(s).

-- This is really a good idea. We added Table 3 containing parameters for simulation.

- When I read the 1st paragraph of the Simulation section, I was expecting to see how changes under the three scenarios and a result that will show under which scenario increases most.

-- This is a valid point. The trend over time in $a^{\dagger}$ and $e^{o}$ would be helpful for a better understanding of the dynamics of other measures based $a^{\dagger}$. Hence, we added the plots of $e^{o}$ and $a^{\dagger}$ in Figure 3.

- You also mention “faster progress above threshold age ”. But what is the value of this threshold age at baseline? And how did the $a^{\dagger}$changed under the 3 scenarios. Given that $e^{\dagger}$, $e_{c}^{\dagger}$, $e_{e}^{\dagger}$ and REC depend on it is important to document its changes under the different scenarios.

-- Thanks. We added a plot for $a^{\dagger}$ in Figure 3 such that the readers could have a clear impression on the trend in threshold age.

- What do the simulated years exactly represent? The way I was expecting the simulations to go is as follow: (1) I have function at and from to mortality declined faster at younger than older ages (2) Look at how this decline affected $e^{\dagger}$, $e_{c}^{\dagger}$, $e_{e}^{\dagger}$and REC. I am guessing that you have repeated this process several times, given the trends in Figure 4. If this is the case, then Figure 4 green line shows trends in $e^{\dagger}$, $e_{c}^{\dagger}$, $e_{e}^{\dagger}$ and REC if mortality declines faster below year after year after year.

-- The simulation conducted in this study follows a standard process. As you said, the simulated years are t=0, 1, 2, … and so on. For each year, mortality rates decline by $\rho(x)$, which is given by the scenarios, and the corresponding $e^{\dagger}$, $e_{c}^{\dagger}$, $e_{e}^{\dagger}$and REC are calculated. The time trend of them are plotted in Figure 3.

Here are my more detailed comments:

- p. 3, line 46: define entropy

-- Thanks. We added a definition to entropy where it appears first time.

- p. 3, line 58: “In a country with increasing younger adult mortality (because of wars or political distress), the compression component can rise and increase lifespan disparity.” My understanding is that compression component declines (i.e. deaths are less compressed) if $\rho\left( x \right)$mortality at younger adult ages increases. I think here you mean the “compression component declines” or compression “component becomes less important”.

-- Thanks. We double-checked the text, finding the sentence is correct.The compression component, and the entire lifespan disparity, will decrease due to mortality reductions. On the contrary, when the mortality rates, particularly at younger ages, increase, the compression component (lifespan disparity) and will increase. This is what happened to Russian men in 1990-1995.

- p. 5, line 89: “entropy less than one”. On p. 3, line 46 “entropy bigger than one” .

-- Thanks for pointing out this. We corrected it.

- p. 8, line 151: “The expansion component can …”, confusing. It seem that in the sentence above you say the opposite.

-- Thanks for pointing out this. We reworded that sentence as “But if the progress of morality improvement among older people is sufficiently great, then the increase in the expansion component can outweigh the decrease in the compression component and the REC will rise.”

- p. 9, line 175: “g(x,t) indicates how efficiently a decrease in …”. What do you mean by efficiently.

- p.9 line 186: “low efficiency”. Explain, why is it considered low efficiency? How much the needs to change in order for the old-age mortality decline to be considered efficient?

-- For the above two points, we added an explanation for “efficiency” and the like: “g(x,t) indicates how much $e^{\dagger}$ will change given a decrease in mortality at age x, that is, the efficiency of mortality reductions in changing $e^{\dagger}$.”

- p. 10: line 196-197: “(5) represents the decrease in the compression component” … “and the second the increase in the expansion component”. Why automatically decrease and increase? You previously mentioned, that the compression component can decline under particular mortality conditions. Why not changes in the compression and expansion component.

-- Thanks. Another reviewer also pointed out this problem. We changed the sentence to “represents the change in the compression component and the second the change in the expansion component.”

- p. 14 line 287-288: Highlight that the REC is under 1 in Figure 3. The value of the REC is important as it is this value that indicates which component is stronger. Important to mention it when describing the Figure.

-- Thanks for your suggestions. We added the REC values in Figure 3, and as you suggested, we mentioned them in the main text.

- p.15 and p.16: again mention that the REC is below 1 and what does this mean (i.e. REC below 1 indicates that the compression is stronger than the expansion) when talking about Figures 4 and 5. An increase in the REC towards 1, indicates that the expansion component is increasing. You do not mention this when describing Figure 4 and 5.

-- Thanks. We realized that the REC cannot be greater than one if mortality rises with age. So in simulations and empirical results we see none of the REC exceed 0.8. Hence, all statements like “the REC is greater one” or “the expansion component outweighs the compression component” are deleted or rephrased to increase in relative importance.

- Discussion section: I would have liked some possible explanations on why the compression component is always bigger than the expansion component, for both cohorts and periods. Do you think that this is due to the fact that $a^{\dagger}$ depends on life expectancy at age x (appears in the function?) Could this be a limitation of the indicator because is highly sensitive to mortality changes at younger than older ages? Also, how does $a^{\dagger}$,$e^{\dagger}$, $e_{c}^{\dagger}$, $e_{e}^{\dagger}$ and REC change with different values of x, i.e. x=0, x=10, x=20? I assume that for the entire manuscript you chose x=0.

-- Thanks for raising a very interesting question. The REC is constructed based on $a^{\dagger}$ that is usually a few years lower than life expectancy at birth, the age interval with g(x)<0 is much larger than that with g(x)>0. Hence, the compression component is greater than the expansion component. This also implies that the REC, as you said, is more likely to be affected by mortality changes at younger ages. But we are not sure if this could be a limitation of the REC because many other measures of lifespan disparity such as $a^{\dagger}$, variance in age at death, or Gini coefficient, etc., are also sensitive to changes in mortality at younger ages, though they have somewhat different threshold ages (see van Raalte and Caswell 2013). The sensitivity to young-age mortality seems a feature regarding the age distribution of deaths for human mortality. Besides, the REC is designed to reflect the change in the relative importance of expansion to compression, and so its sensitivity to young-age mortality is part of what it can and should capture.

In this study, we mainly focus on the entire age range. We agree with you that the trajectories of the REC with x=10, 20, … may reveal some interesting results. Indeed, we are now working on some extension on the REC, including the REC(x) will

References:

van Raalte AA, Caswell H. Perturbation analysis of indices of lifespan variability. Demography. 2013;50(5):1615-40.
