# Supplementary material for: The ratio of expansion to compression: A new measure of lifespan disparity
Source: PLoS One. 2020 Dec 14;15(12):e0243482. doi: 10.1371/journal.pone.0243482 (PMC7735627; doi:10.1371/journal.pone.0243482)
Supplement: Attachment — Submitted filename: Response to Reviewers.docx [file pone.0243482.s005.docx]

Dear Viorela Diaconu, dear Ugofilippo Basellini,

Thank you very much for your helpful comments and suggestions. Your comments so far have encouraged us to think hard and express clearly, which greatly improves the manuscript.

Cordially,

Qiang

Reviewer #1: Dear authors,

Thank you for adequately addressing my previous comments and suggestions on your manuscript. While reading the revised version of your manuscript, I found that the newly added sections need some improvement.

Comment 1:
I find that the explanations of the different scenarios under which REC increases/decreases are not clear enough. My understanding from the explanations provided later on for Fig. 1, is that REC and its components depend on the mortality changes before and after the threshold age. I think it is important to mention this in the scenarios. For instance, when mortality at younger ages increases and mortality at older ages declines (?), increases (?), this is what happens to the REC. More precisely:

Line 146: “The REC can increase in two cases. The first case is a rise in young-age mortality […]”. In this first case, changes that occur in mortality at older ages do not matter?
-- Thanks for pointing out this. Indeed, any change of the REC will involve both the compression and expansion components. Hence, we rewrote the part as follows:

“The first case is a rise in young-age mortality, which increases the compression component. Moreover, the increase in young-age mortality lowers the threshold age and widens the interval $\left[ a^{\dagger},\omega\right]$, thereby further augmenting the expansion component. When the expansion term rises by more than the compression term, the REC increases.”

Line 150: “In the second case, when old-age mortality has a faster decline […]”. Faster decline than what? I assume than at younger ages.
-- Thanks. We rewrote the sentence to “when old-age mortality declines faster than young-age mortality.”

Also, the REC can increase in two cases. Can it not increase? Is there any doubt on the fact that it increases? Does it depend on something else? For me, the “can increase” formulation suggests that there is a possibility that under some condition it will not increase.
-- This is a valid point. As shown in the decomposition analysis and the simulation results, the REC is likely to be constant over time wherever the relatively change of the compression component is the same as that of the expansion component. The sentence below Eq (8) is another kind of expression of the same meaning. We hope this will be clear enough.

Line 155: I will not mention in this paragraph the decline in REC given that you explain at line 168 the scenarios under which REC declines.

Line 171: In the second case […]. What are the mortality conditions under this case?
-- We agree that the part is not very clear, so we revised it as follows:

“In the second case, the expansion component decreases due to a fast rise in threshold age, which squeeze the interval$[a^{\dagger}, \omega]$, accompanied by the relatively slow pace of old-age mortality reduction, while the compression component may decrease slowly or even increase; thus, the REC decreases.”

Comment 2:
Line 60-62: "In a country with increasing younger adult mortality (because of wars or political distress), the compression component can rise and increase lifespan disparity".
Line 146: "The first case is a rise in young-age mortality, with a corresponding decrease in life expectancy and threshold age. The compression term may decrease because the interval [0, †] decreases".
Following an increase in mortality at younger ages, the compression component can increase (lines 60-62), but may decreases (lines 146). Which one is it?

-- Thanks. As noted in the above reply, we re-wrote the paragraph about the “The first case…”, to make them consistent. Please see above response and the revision for details.

Comment 3:
Figure 2: legend of colors doesn’t show

Reviewer #2: I would like to thank the authors for addressing all the comments and suggestions that I raised in the first round of reviews. I believe that the manuscript has improved and gained in clarity with respect to the previous draft. Also, I thank the authors for acknowledging my name in the Acknowledgment section, I really appreciated it and I am glad that my comments helped them to improve the manuscript.

I have three final minor comments for the authors:
- line 148: change "morality" with "mortality"
- line 190: "The disparity will rise when \dot{e}^{\dagger}_e is greater than \dot{e}^{\dagger}_c and viceversa". There is a minus missing in \dot{e}^{\dagger}_c
-- Thanks for pointing out the typos. We corrected them in the revision.

- Figure 2: please add back the legend of the years for easier readability

-- Many thanks for this suggestion. We added the legend of the years in Figure 2, which looks much clearer.
